# Reproduction and beyond, kisspeptin in ruminants

**DOI:** 10.1186/s40104-015-0021-4

**Published:** 2015-05-28

**Authors:** Joseph A. Daniel, Chad D. Foradori, Brian K. Whitlock, James L. Sartin

**Affiliations:** Department of Animal Science, Berry College, Mt. Berry, GA 30149 USA; Department of Anatomy, Physiology & Pharmacology, Auburn University, Auburn, AL 36849 USA; Department of Large Animal Clinical Sciences, University of Tennessee, Knoxville, TN 37996 USA

**Keywords:** Growth hormone, Leptin, Luteinizing, Neuropeptide Y, POMC

## Abstract

Kisspeptin (Kp) is synthesized in the arcuate nucleus and preoptic area of the hypothalamus and is a regulator of gonadotropin releasing hormone in the hypothalamus. In addition, Kp may regulate additional functions such as increased neuropeptide Y gene expression and reduced proopiomelanocortin (POMC) gene expression in sheep. Other studies have found a role for Kp to release growth hormone (GH), prolactin and luteinizing hormone (LH) from cattle, rat and monkey pituitary cells. Intravenous injection of Kp stimulated release LH, GH, prolactin and follicle stimulating hormone in some experiments in cattle and sheep, but other studies have failed to find an effect of peripheral injection of Kp on GH release. Recent studies indicate that Kp can stimulate GH release after intracerebroventricular injection in sheep at doses that do not release GH after intravenous injection. These studies suggest that Kp may have a role in regulation of both reproduction and metabolism in sheep. Since GH plays a role in luteal development, it is tempting to speculate that the ability of Kp to release GH and LH is related to normal control of reproduction.

## Introduction

Kisspeptin (Kp), also known as metastin, was first discovered and noted for its role in the inhibition of cancer cell metastasis. However, it was discovered to also stimulate gonadotropin releasing hormone (GnRH) release and subsequent secretion of luteinizing hormone. Neuroendocrine control of reproduction in ruminants culminates in the secretion of luteinizing hormone (LH) from the anterior pituitary. There has been much interest in Kp’s role in regulation of reproduction in a number of species including ruminants. More recently, Kp has been implicated in the integration of metabolic control of reproduction. This review will briefly summarize KP action on reproduction in ruminants and focus on recent discoveries of Kp action beyond reproduction control in ruminants.

## Review

### Kisspeptin action on luteinizing hormone and gonadotropin

Kisspeptin clearly stimulates a release of GnRH and subsequent secretion of LH. Intravenous administration of Kp-10 to ovariectomized (OVX) ewes stimulated increased circulating concentrations of LH and increased GnRH concentrations in the cerebrospinal fluid [1]. Central administration of Kp-10 increased GnRH concentrations in the cerebrospinal fluid and increased LH concentrations in the plasma of sheep [2]. Additionally, Kp-10 increased circulating concentrations of LH in prepubertal male and female Japanese Black calves [3]. Kisspeptin-10 also stimulated increased circulating concentrations of LH in Holstein cows and ovariectomized Jersey cows, and interestingly the sensitivity of LH to exogenous Kp-10 stimulation seems to be enhanced with lactation [4, 5]. Central administration of the Kp antagonist, peptide 234, to ewes reduced LH pulse amplitude to the point of precluding determination of pulse frequency and reduced mean LH concentrations [6]. Central administration of p-271, another Kp receptor (Kiss1R) antagonist, also inhibited pulsatile LH concentrations in ovariectomized ewes [7]. Kisspeptin expression is regulated by steroids as the number of Kp positive cells in the ARC are increased following OVX compared to intact ewes, the opposite being found in the preoptic area (POA) Kp neurons [8]. Furthermore, the number of Kp positive cells in the arcuate nucleus (ARC) is reduced in ovariectomized ewes by treatment with estrogen or progesterone [8]. Additionally, the majority of Kp positive cells in the ARC also coexpressed the progesterone receptor [8]. Interestingly, single nucleotide polymorphisms in the *Kiss1* gene were associated with increased litter size in goats [9].

### Kisspeptin and the luteinizing hormone surge

Kisspeptin appears to have a role in generation of the LH surge to stimulate ovulation. Constant iv infusion of Kp-10 for 8 h beginning 30 h after progesterone withdrawal stimulated an earlier LH surge and an earlier increase in circulating concentrations of progesterone than in ewes treated with vehicle [1]. Additionally, IV infusion of Kp-10 also stimulates a surge of LH in anestrous ewes [10]. Blockage of Kp action with the Kiss1R antagonist, p-271, attenuated an estradiol induced LH surge [8].

The action of Kp on LH in sheep appears to be via an effect on GnRH release from the hypothalamus and not direct action of Kp on pituitary gonadotropes. Cultured ovine pituitary cells can respond to Kp treatment with increased release of LH, but hypothalamo-pituitary-disconnected ewes do not respond to Kp-10 treatment with increased circulating concentrations of LH nor do hypophysial portal concentrations of Kp correspond with LH pulsatility and the LH surge [11]. Furthermore, expression of Kp (both number of Kp positive cells and level of expression per cell) was increased in caudal ARC during the late follicular phase of ewes [12]. Smith et al. [13] also observed an increase in the number of Kiss1 mRNA positive cells in the middle and caudal ARC as well as the POA during the late follicular phase. Additionally, the percentage of Kp positive cells expressing Fos was increased by positive estradiol feedback in the middle and caudal ARC [13]. Kisspeptin neurons in the POA showed high levels of Fos activation at the time of the LH surge [14]. The proportion of Kp neurons showing Fos activation was positively correlated with the percentage of GnRH neurons expressing Fos activation [14]. However, very few ARC Kp neurons showed Fos activation around the time of the LH surge [15]. Thus, Kp is clearly involved in generation of the GnRH, and consequentially the LH, surge, although it is not clear if Kp neurons in the ARC or POA area are more important in generating the LH surge.

### Kisspeptin action in seasonally anestrous animals

The Kp response to steroid and nonsteroid cues is altered during the nonbreeding season to result in seasonal anestrous in sheep. In ewes, infusion of Kp-10 IV stimulated ovulation during the nonbreeding season [1]. Kisspeptin expression is higher in the breeding season than the nonbreeding season in sheep, and there is an increase in Kp contacts with GnRH neurons during the breeding season [12]. The number of Kp positive cells in the ARC is also higher during the breeding season than the nonbreeding season of ovariectomized ewes [8]. Additionally, the number Kp positive neurons in the ARC and the percentage of neurons positive for Kp in both the ARC and preoptic area increased in OVX, estradiol treated ewes following the transition to short day exposure [16]. The GnRH and LH response to Kp-10 is greater in seasonally anestrous ewes than in luteal ewes during the breeding season [17]. Additionally, expression of the Kp receptor, Kiss1r, mRNA in GnRH neurons is higher during the nonbreeding season than during the breeding season in ewes, and Kiss1r mRNA expression in GnRH neurons is reduced by Kp-10 treatment of ewes during the non-breeding season but not by steroid treatment of OVX ewes [17]. These results suggest alterations in Kp production or release is involved in the seasonal regulation of reproduction in sheep.

Introduction of a ram to seasonally anestrous ewes isolated from rams for at least one month will induce pulsatile LH secretion and can cause ovulation outside of the breeding season [18]. Kisspeptin has a role in this response. Indeed, De Bond et al. [19] utilized the Kp antagonist (p-271) to demonstrate Kp action is necessary for the seasonally anestrous ewes to respond to ram introduction with increased LH. Furthermore, ram introduction also increased the number of Kp positive neurons and *Kiss 1* mRNA expression in cells in the rostral ARC [19]. Interestingly, *Tac2* mRNA, encoding for neurokinin B, was readily detectable in cells with *Kiss 1* mRNA, but was decreased in rostral ARC cells following ram introduction [19].

### Kisspeptin action in prepubertal animals

Puberty in the ruminant is initiated by a decrease in negative feedback inhibition of LH by estradiol. Kisspeptin and neurokinin B, which is co-expressed with Kp in a number of hypothalamic cells, may play a role in initiation of puberty in ruminants. Injection of senktide, a neurokinin B agonist, in prepubertal ewes was immediately followed by a LH pulse [20]. However, the number of neurokinin B cells in the ARC was not different in prepubertal and post puberty ewes, although there was an increase following ovariectomy [20]. The number of Kp positive cells in the ARC increased following puberty in intact ewes and was also increased following ovariectomy in prepubertal ewes [20]. Additionally, Redmond et al. [21] observed the number of *Kiss 1* positive cells in the POA increased from 25 to 35 weeks of age in ewe lambs, although the number of *Kiss 1* positive cells did not appear to be related to increases in LH pulse frequency. However, Redmond et al. [21] did report an increase in *Kiss 1* positive cells in the middle ARC that was associated with increased frequency of LH pulses. Furthermore, the percentage of GnRH neurons in the POA with Kp positive close contacts was higher in post puberty in ewes and increased following ovariectomy in prepubertal ewes [20]. Hourly intravenous treatment with Kp is capable of inducing a LH surge followed by elevated concentrations of progesterone, suggesting ovulation, in prepubertal ewe lambs [22]. Thus, Kp has a definite role in initiation of puberty in the sheep, but only in a defined window of receptivity.

### Non-Reproductive roles for Kisspeptin

#### Pituitary actions of kisspeptin

There is circumstantial evidence of possible actions of Kp at the level of the pituitary in sheep, possibly actions not oriented towards regulation of LH. For example, in sheep the median eminence contains neuron terminals with specific staining for Kp [23]. Kisspeptin is also released into the portal vein circulation of sheep [11] however the timing of Kp pulses was concurrent with or follow the LH pulses. This may suggest that Kp is regulating some pituitary function other than stimulating LH via GnRH action. Kotani et al. [24] described the presence of Kiss1R in human pituitary followed by studies in sheep which found the presence of Kp receptor mRNA in gonadotropes, lactotropes and somatotropes [8]. The same year, Kadokawa et al. [25] incubated pituitary cells isolated from bovine pituitaries and cultured in the presence of variable doses of Kp-10. The pituitary cells responded to the direct application of Kp-10 in two hours with a dose-dependent increase in secretion of growth hormone (GH) and prolactin. In addition, Gutierez-Pascal et al. [26] found similar results using pituitary cells isolated from rats following treatment with Kp-10 for 30 min or 4 h. The rat pituitary cells also demonstrated an increase in intracellular Ca^++^ that occurred in 10 % of cultured cells. Moreover, in nonhuman primate pituitary cell culture, Kp-10 stimulated both GH and LH release through extracellular Ca^++^ entry, phospholipase C, protein kinase C, MAPK, and by additional intracellular Ca^++^ mobilization [27]. These data collectively suggests an intrapituitary signaling system for modification of GH release and perhaps prolactin.

Peripheral administration of Kp-10 to prepubertal heifers increased circulating concentrations of GH [28], suggesting a physiological role to regulate GH from the pituitary. Moreover, Sébert et al. [10] found that intravenous infusion of Kp-10 to seasonally acyclic ewes produced a surge of LH that was accompanied by a smaller but significant rise in plasma concentrations of both FSH and GH. However, others have reported Kp administration did not alter circulating concentrations of GH in prepubertal gilts [29], Kp-10 administration did not alter circulating concentrations of GH in goats [30], lactating dairy cows [5], or prepubertal male or female cattle [3], and Kp-10 administration did not alter plasma GH, prolactin, TSH and cortisol in rhesus monkeys [31]. In OVX cows, intravenous administration of Kp-10 stimulated increased circulating concentrations of LH regardless of supplemental steroid treatment, but only increased circulating concentrations of GH in cows treated with pharmacologic doses of estradiol cypionate and/or progesterone [32]. Even in the presence of the steroids, the GH pulse was only 2–3 fold higher than baseline plasma concentrations with a duration of only 10 min. Thus actions of peripheral Kp to regulate GH release are inconsistent and species dependent. In view of the clear actions of Kp to release GH *in vitro*, as well as release GH after intravenous injection under some conditions, it can be concluded that Kp has the potential to modify GH through actions at the pituitary, but is probably not a major direct regulator of GH release.

#### Hypothalamic actions of Kisspeptin

**Fig. 1:**
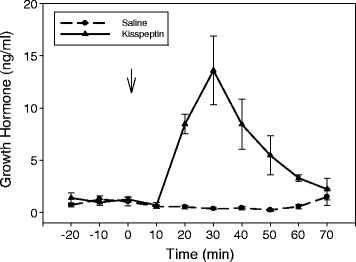
Effect of intracerebroventricular injection of kisspeptin (200pmol/Kg BW) on circulating concentrations of growth hormone in sheep. (unpublished data)

Infusion of Kp-10 via the lateral ventricle of sheep resulted in an increase in ARC neuropeptide Y (NPY) gene expression and a decrease in proopiomelanocortin gene expression [33]. Since leptin receptors were found in Kp-positive neurons in sheep, this finding suggested a mechanism to coordinate reproduction and metabolic control in sheep. Interestingly, a study in NPY-GFP transgenic mice found that Kiss1r mRNA was produced in NPY neurons [34]. In a hypothalamic cell line, Kim et al. [34] also found that Kp-10 directly regulates NPY neuron synthesis and release, providing more credence to a possible link for Kp to metabolic control. Since NPY is known to control GH release in ruminants [35], the results of these experiments with NPY suggest that Kp could regulate GH release through the hypothalamus. In an experiment to test this hypothesis, doses of Kp-10 of 100, 200 or 1,000 pmol/kg BW of Kp-10 were administered intravenously to ovariectomized sheep. There was an expected response to increase plasma LH concentrations, but even at the highest dose of Kp-10, there were no effects on circulating concentrations of GH. Central administration of Kp-10 at 100 or 200 pmol/kg BW increased GH release as well as the expected release of LH [4]. An example of this effect of Kp to release GH is found in Fig. 1 (unpublished data). Thus Kp, working via hypothalamic mechanisms, provides a strong stimulus to increase GH in addition to its well-known effects on LH release in sheep.

At present there have been no published findings regarding mechanism or the physiological significance for Kp in regulating GH. The link between Kp and NPY [33] and the evidence that NPY releases GH in ruminants [35]), suggests a possible mechanism for GH regulation by Kp, though to date there have been no direct studies of this proposed pathway. In terms of physiological relevance, the findings that both LH and GH are needed for normal luteal growth in sheep suggests that GH release may be linked to reproductive success [36]. Therefore it is tempting to speculate that in addition to direct mechanism regulating GnRH and hence LH, Kp may also regulate GH through hypothalamic mechanisms and this GH regulation may be a critical component of normal reproduction in sheep. However, this hypothesis has not yet been directly examined.

## Conclusions

These studies confirm a link between Kp and metabolic regulatory systems. Since adequate nutrition and GH are both needed for reproductive success, the connection of Kp to metabolic and GH systems may be a critical component of normal reproduction and should be examined in more depth.
